# Numerical and experimental dataset for a retrofitted data center

**DOI:** 10.1016/j.dib.2022.108587

**Published:** 2022-09-11

**Authors:** Mustafa Kuzay, Aras Dogan, Sibel Yilmaz, Oguzhan Herkiloglu, Ali Serdar Atalay, Atilla Cemberci, Cagatay Yilmaz, Ender Demirel

**Affiliations:** a Design and Simulation Technologies Inc., Eskisehir 26480, Turkey; b Radius Solution Center, Kisikli, Bahar Arkasi Street, No:11, Camlica, Uskudar, Istanbul 34692, Turkey; c Lande Industrial Metal Products Inc. Co. Organized Industrial Zone, 20th Street, No: 14, Eskisehir, Turkey; d Eskisehir Osmangazi University, Eskisehir 26480, Turkey

**Keywords:** Data center, Retrofitting, CFD, OpenFOAM, Temperature measurement

## Abstract

This article provides experimental and numerical data for the flow and thermal distributions inside an air-cooled data center. The experimental data contains the exhaust temperature profile obtained from an experimental campaign and the numerical data contains OpenFOAM and script files for the simulation of the thermal structure based on the experimental study. Experimental measurements were conducted using temperature sensors located at the rear of the rack under a working scenario of 2 kW. Publically available experimental data, numerical model and results can be used for the validation of numerical models under the thermal scenario given in the present study. Flow and thermal structures inside the data center are exhibited using the validated numerical model.

**Specifications Table**

**Table:** 

Subject:	Mechanical Engineering
Specific subject area:	Data center, Temperature measurement, Thermal Structure, OpenFOAM, Cooling Efficiency.
Type of data:	Tables Figures OpenFOAM Cases Experimental Data
How the data were acquired:	• 11 American Power Conversion (APC) [1] temperature sensors were used for the measurement of exhaust temperatures under a working scenario with 2 kW. • Temperature sensors were located at the top of the rack door for the optimum measurement performance. • Temperature sensors were connected to the NetBotz rack monitor to collect temperature data during two hours with a sampling frequency of 0.1 Hz. • Numerical data was generated using a validated open source numerical model based on OpenFOAM libraries.
Data format:	Raw Analyzed
Description of data collection:	Experimental studies were carried out under a defined working scenario of 2 kW for both previous and retrofitted designs. Numerical model of the data center was prepared using OpenFOAM libraries for the modeling of server components and cooling device inside the data center. Numerical simulations are performed with parallel computing on a High Performance Computing (HPC) center.
Data source location:	• **Institution:** Radius Solution Center • **City/Town/Region:** Camlica/Uskudar/Istanbul • **Country:** Turkey • **Latitude and longitude (and GPS coordinates, if possible) for collected samples/data:** 41°01′29.6"N 29°04′43.2"E
Data accessibility:	**Repository name:** Numerical and experimental dataset for an air-cooled data center **Data identification number:**10.5281/zenodo.7035829
Related research article:	M. Kuzay, A. Dogan, S. Yilmaz, O. Herkiloglu, A.S. Atalay, C. Yilmaz, E. Demirel, Retrofitting of an air-cooled data center for energy efficiency, Case Studies in Thermal Engineering (2022), 36, 102228. https://doi.org/10.1016/j.csite.2022.102228.

## Value of the Data

• Data center managers and authorities will benefit from the experimental and numerical data for the validation of a numerical model on an operating data center.
• The numerical data provides OpenFOAM files and scripts for the simulation of flow and thermal structures inside an air-cooled data center.

## 1 Data Description

**Fig 1:**
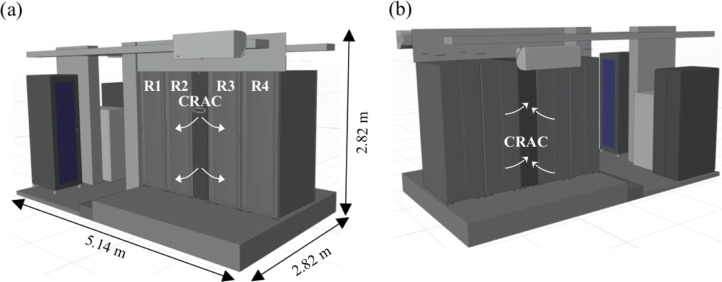
Three-dimensional geometry of the Bitnet data center: (a) front and (b) back views.

**Fig 2:**
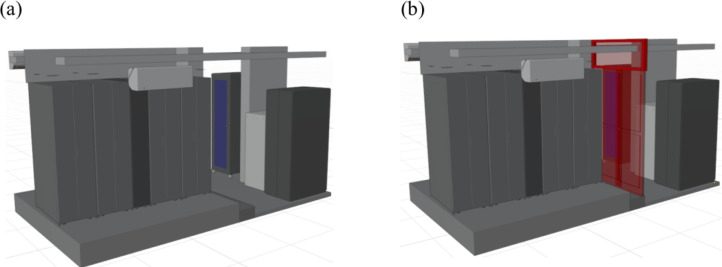
Three-dimensional geometry of Bitnet data center back views: (a) previous and (b) retrofitted designs.

The data reported in this study contains results of the experimental and numerical studies conducted in the Radius Solution Center (Fig. 1). The data center is comprised of four racks and an in-row cooling unit to supply cold air. Numerical simulation results show that thermal and cooling efficiencies of the data center are low [2]. In order to increase the cooling efficiency of the data center, hot and cold aisles were isolated using a sliding door at the rear of the racks (Fig. 2). Experimental data contains temperature measurements for previous and retrofitted designs under a 2 kW working scenario described in Table 1.

**Table 1:** Rack layout and working scenario for the experimental study.

Rack	First Row	Height [U]	Power Consumption [W]	Flow Rate (m^3^/s)
1	38	1	84	0.00740
2	27	2	144	0.02390
2	29	2	144	0.02390
3	25	2	154	0.03300
3	27	2	322	0.06700
3	29	2	196	0.04060
3	31	2	112	0.03300
3	35	2	210	0.01900
4	34	2	468	0.04450
4	38	2	165	0.01400

**Fig 3:**
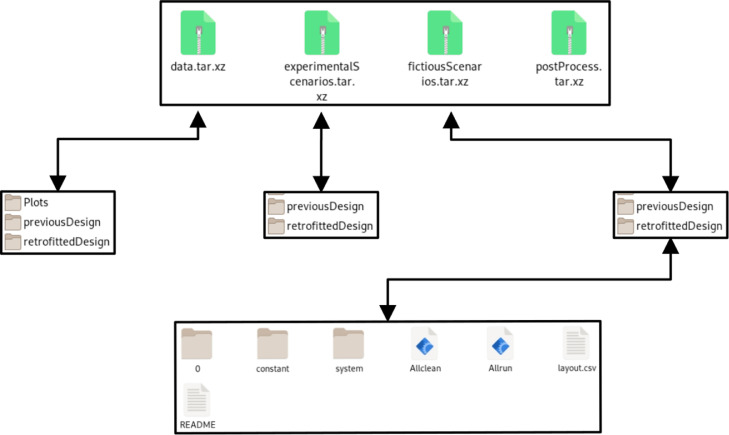
Structure of the experimental and numerical data.

Structure of the dataset is shown in Fig. 3. The “data.tar.xz” file contains the experimental data related with the time variations of exhaust temperature and flow rate of the in-row cooling unit. The “experimentalScenarios.tar.xz” file contains OpenFOAM [6] files and scripts for the simulation of flow and thermal structures under the same thermal conditions as in the experimental studies for previous and retrofitted designs.

The “fictiousScenarios.tar.xz” file contains OpenFOAM files of previous and retrofitted designs in Fig. 2, for a fictitious working scenario of 15.5 kW. Where, *fictiousScenarios.tar.xz/privousDesign* and *fictiousScenarios.tar.xz/retrofittedDesign* folders contain following subfolders and files:

• Initial and boundary conditions for temperature, velocity, dynamic pressure, turbulent kinetic energy, turbulent kinetic energy dissipation rate, alphat, turbulent viscosity, pressure and turbulence specific dissipation are contained in the *0* folder.
• The *constant* folder contains following folders and files: The p*olyMesh* folder contains files created by *blockMeshDict* and *snappyHexMeshDict* utilities for the generation of the geometry, *fvOptions* and *fvOptionsInclude* files for the definitions of heat sources inside the servers, *g* file for the definition of the gravitational acceleration, *momentumTransport* file for the turbulence closure model, *pRef* file for the reference pressure, *thermophysicalProperties* file for the definition of the thermodynamic features of the air. Although layouts of the previous and retrofitted designs are identical, *polyMesh* folders are slightly different due to the modification of the room by locating a baffle at the rear of the racks.
• The *system* folder contains following files: The *controlDict* file is used to specify key state controls such as timing information, write format and specifying optional libraries that can be loaded at runtime. Variables are specified in the *DCMetrics* file for the calculation of the efficiency metrics by defining active servers, maximum and minimum allowable temperatures and maximum and minimum recommended temperatures. The *decomposeParDict* file contains features of the parallel computing such as method and number of subdomains. The *fvSchemes* file sets up numerical schemes for the discretization of the time variation, convective and diffusive terms in the governing equations. The *fvSolution* file contains features of the matrix solver and residual controls for each variable. The cell zones are defined according to the features defined in the *topoSetDict* file to assign heat sources to the servers.

The “postProcess.tar.xz” file in Fig. 3 contains OpenFOAM files of the time-averaged results and paraview files for the front and back views of the data center. User can open these files for the post processing of the results without any adjustment such as viewpoint and coloring range for the temperature field. Rack layout and working scenario, comparison of experimental and numerical results at the measurement locations and numerical results are given In *FiguresAndTables* folder.

Mesh independence study was carried out using *Inlet.py* and *Exhaust.py* files contained in the *fictiousScenarios/retrofittedDesign/results/figures* folder. The experimental data contained in the *Inlet.csv* and *Exhaust.csv* files can be plotted using these Python codes [7]. Inlet and exhaust temperature data were monitored at 5 different locations at the front and rear of the Rack 3 during mesh independence study. Distributions of the inlet and exhaust temperatures are given in Fig. 1. png and Fig. 2. png, respectively, for the mesh independence study. The *layout.csv* file contains the information related with the rack, first row, height (U), type, power consumption and flow values of the servers.

## 2 Experimental Design, Materials and Methods

### 2.1 Experimental Study

**Fig 4:**
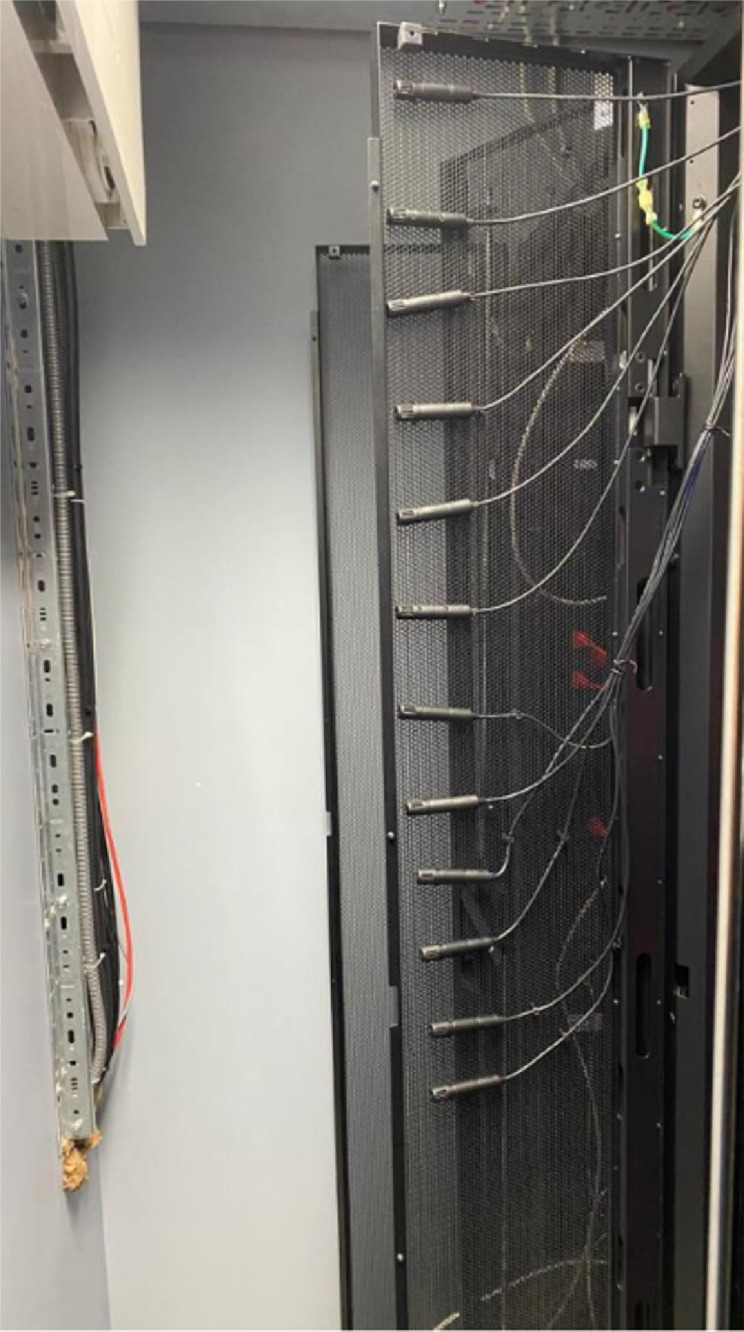
Temperature sensors at the rear of the rack.

Experimental studies were conducted for previous and retrofitted designs (Fig. 2) in terms of temperature measurements at the rear of the rack using 11 APC temperature sensors. It is recommended by the manufacturer to install sensors near the top of the rack door for the optimum measurement performance since bottom of the rack door does not accurately represent the air temperature inside the data center [1]. As shown in Fig. 4, the APC temperature sensors were placed at the upper part of the rack door. Temperature sensors were located at the rear of the Rack 3 and connected to the NetBotz rack monitor to collect temperature with a sampling frequency of 0.1 Hz.

### 2.2 Numerical Study

Numerical simulations are performed using an open source numerical model for the working scenario described in *layout.csv* for each case. The buoyantPimpleFoam solver is used for the simulation of buoyancy driven turbulent flow inside the data center. The Shear Stress Transport (SST) turbulent closure model is used to account for the negative pressure gradients and boundary layer effects near the walls [2,4]. Server components are modeled as open-box model, in which power consumption can be applied as a heat source and flow rate of the air passing through the sever can be adjusted as lt/s according to the corresponding power consumption [1,2]. Another approach is the black-box approach for the modeling of server and cooling units, where jump conditions are applied at the inlet and outlet of the server according to the corresponding power consumption. The CRAC unit is modeled using a black-box approach in which the inlet and outlet boundary conditions are used without solving the internal flow field [2], [3] to reduce simulation durations. The porous approach is used for the modeling of server components and perforated rack doors. The inertia and viscous resistance by the server components are modeled using Darcy-Forchheimer porosity model [5]. Porosity coefficients were determined from the geometrical features of the components. Details of the numerical model can be found in [2].

Mean values of the exhaust temperatures obtained from the experimental studies are given in the *FiguresAndTables* folder of the data set for previous and retrofitted designs. Numerical simulation results are compared with the experimental data (in the *FiguresAndTables* folder of the data set) in terms of the exhaust temperature profile. A good agreement observed between numerical and experimental data shows that the present numerical model can accurately capture thermal structure in a data center. The maximum and average errors are calculated as 1.1°C and 0.56°C, respectively, for the previous design. On the other hand, the maximum and mean errors are calculated as 1.6°C and 0.47°C, respectively, for the retrofitted design.

The volumetric distribution of the local temperature (in the *FiguresAndTables* folder of the data set) clearly shows hot regions originating from the recirculation of hot flows at the top of the data center. Hot regions occupy almost half of the volume at the rear part of the data center. The present numerical model can clearly reveal thermal issues in a data center and design alternatives can be investigated and developed to suppress predicted issues for the efficiency enhancement.

## Ethics Statements

This work did not include work involved with human subjects, animal experiments or data collected from social media platforms.

## CRediT authorship contribution statement

**Mustafa Kuzay:** Methodology, Software, Validation, Visualization, Formal analysis. **Aras Dogan:** Methodology, Software, Validation, Visualization, Formal analysis. **Sibel Yilmaz:** Methodology, Software, Validation, Visualization, Formal analysis. **Oguzhan Herkiloglu:** Data curation, Methodology. **Ali Serdar Atalay:** Resources. **Atilla Cemberci:** Resources. **Cagatay Yilmaz:** Project administration, Funding acquisition. **Ender Demirel:** Conceptualization, Writing – review & editing, Investigation, Supervision.

## Declaration of Competing Interest

The authors declare that they have no known competing financial interests or personal relationships that could have appeared to influence the work reported in this paper.

## Data Availability

Numerical and experimental dataset for an air-cooled data center (Original data) (Zenodo).
